# Prolonged antibiotic prophylaxis after pancreatoduodenectomy: systematic review and meta-analysis

**DOI:** 10.1093/bjs/znad213

**Published:** 2023-07-13

**Authors:** Daphne H M Droogh, Jesse V Groen, Mark G J de Boer, Joffrey van Prehn, Hein Putter, Bert A Bonsing, Casper H J van Eijck, Alexander L Vahrmeijer, Hjalmar C van Santvoort, Bas Groot Koerkamp, J Sven D Mieog

**Affiliations:** Department of Surgery, Leiden University Medical Centre, Leiden, the Netherlands; Department of Surgery, Leiden University Medical Centre, Leiden, the Netherlands; Departments of Infectious Diseases and Clinical Epidemiology, Leiden University Medical Centre, Leiden, the Netherlands; Department of Medical Microbiology, Leiden University Medical Centre, Leiden, the Netherlands; Department of Biomedical Data Sciences, Leiden University Medical Centre, Leiden, the Netherlands; Department of Surgery, Leiden University Medical Centre, Leiden, the Netherlands; Department of Surgery, Erasmus MC Cancer Institute, Rotterdam, the Netherlands; Department of Surgery, Leiden University Medical Centre, Leiden, the Netherlands; Department of Surgery, Regional Academic Cancer Centre Utrecht, Utrecht, the Netherlands; Department of Surgery, Erasmus MC Cancer Institute, Rotterdam, the Netherlands; Department of Surgery, Leiden University Medical Centre, Leiden, the Netherlands

## Abstract

**Background:**

Previous studies have reported conflicting results of prolonged antibiotic prophylaxis on infectious complications after pancreatoduodenectomy. This study evaluated the effect of prolonged antibiotics on surgical-site infections (SSIs) after pancreatoduodenectomy.

**Methods:**

A systematic review and meta-analysis was undertaken of SSIs in patients with perioperative (within 24 h) *versus* prolonged antibiotic (over 24 h) prophylaxis after pancreatoduodenectomy. SSIs were classified as organ/space infections or superficial SSI within 30 days after surgery. ORs were calculated using a Mantel–Haenszel fixed-effect model.

**Results:**

Ten studies were included in the qualitative analysis, of which 8 reporting on 1170 patients were included in the quantitative analysis. The duration of prolonged antibiotic prophylaxis varied between 2 and 10 days after surgery. Four studies reporting on 782 patients showed comparable organ/space infection rates in patients receiving perioperative and prolonged antibiotics (OR 1.35, 95 per cent c.i. 0.94 to 1.93). However, among patients with preoperative biliary drainage (5 studies reporting on 577 patients), organ/space infection rates were lower with prolonged compared with perioperative antibiotics (OR 2.09, 1.43 to 3.07). Three studies (633 patients) demonstrated comparable superficial SSI rates between patients receiving perioperative *versus* prolonged prophylaxis (OR 1.54, 0.97 to 2.44), as well as in patients with preoperative biliary drainage in 4 studies reporting on 431 patients (OR 1.60, 0.89 to 2.88).

**Conclusion:**

Prolonged antibiotic prophylaxis is associated with fewer organ/space infection in patients who undergo preoperative biliary drainage. However, the optimal duration of antibiotic prophylaxis after pancreatoduodenectomy remains to be determined and warrants confirmation in an RCT.

## Teaser

Antibiotic prophylaxis varies substantially between institutes. The effect of prolonged antibiotic prophylaxis seems promising, particularly in patients undergoing pancreatoduodenectomy with contaminated bile. This systematic review and meta-analysis demonstrated a beneficial effect of prolonged antibiotic prophylaxis after pancreatoduodenectomy in patients who had preoperative biliary drainage.

## Introduction

Surgical-site infections (SSIs) and postoperative pancreatic fistulas (POPFs) account for approximately 28–48 per cent of the postoperative morbidity after pancreatoduodenectomy^1,2^. Previous studies^2,3^ demonstrated an association between preoperative biliary drainage, positive bile cultures, and SSIs, and hypothesized that perioperative spillage of contaminated bile may account for the increased rate of SSIs. Moreover, some studies^4–7^ suggested a correlation between abdominal contamination and the development of pancreatic fistula. Hence, optimalization of antibiotic prophylactic regimens might not only reduce the rate of SSIs, but also decrease POPF rates.

The additional benefit of prolonged antibiotic prophylaxis after pancreatoduodenectomy has not been determined. Most studies investigating postoperative antibiotic prophylaxis focused on the effect of tailored prophylaxis, predominantly based on bile cultures obtained before operation^8^. Tailored prophylaxis for each patient has several practical limitations because bile culture results are not available immediately after surgery. Use of standard prolonged antibiotic prophylaxis would be a feasible alternative. The updated enhanced recovery after surgery protocol^9^ states that ‘postoperative “prophylactic” antibiotics are not recommended but may be considered therapeutic in patients with positive bile cultures’. However, the American Society of Health-System Pharmacists, the Infectious Diseases Society of America, the Surgical Infection Society, the Society for Healthcare Epidemiology of America, and the European Centre for Disease Prevention and Control guidelines^10,11^ for perioperative antibiotic prophylaxis recommend against antibiotic prophylaxis prolonged beyond 24 h after abdominal surgery. Consequently, antibiotic regimens vary substantially between institutes, which could imply unnecessary administration of antibiotics, potentially leading to increasing antibiotic resistance^12^.

This systematic review and meta-analysis evaluated the effect of prolonged antibiotic prophylaxis on infectious complications after pancreatoduodenectomy. Additionally, the effect of prolonged antibiotic prophylaxis was studied separately in patients who underwent preoperative biliary drainage.

## Methods

### Literature search and study selection

The literature search included the main terms ‘pancreatoduodenectomy’, ‘antibiotics’, and ‘prophylaxis’, and their related concepts and synonyms (*Appendix S1*). The literature search was performed in PubMed, Embase, Web of Science, the Cochrane library, and Emcare until November 2022. Titles and abstracts were screened independently by two authors for full-text articles written in English investigating prolonged antibiotic prophylaxis after pancreatoduodenectomy. Eligibility criteria for study selection were patients undergoing pancreatoduodenectomy as main subjects, comparison of the duration of postoperative antibiotic prophylaxis (in particular, not only comparison of type of antibiotics), and outcomes related to infectious complications. Case reports, case series, and literature reviews were excluded. This systematic review of the literature was conducted according to the PRISMA statement^13^. The study protocol was registered in PROSPERO (CRD42022321755).

### Data collection

Data extraction was undertaken using a standardized form, including study characteristics, performance of preoperative biliary drainage and acquisition of intraoperative bile cultures, postoperative (infectious) complications, and antibiotic prophylaxis and therapy. Risk of bias was assessed using the Risk Of Bias In Non-randomized Studies—of Interventions (ROBINS-I) tool for cohort studies and the Cochrane tool for randomized trials^14,15^. Studies that were considered to have a serious risk of bias were excluded.

### Outcomes and comparisons

The primary outcome was the rate of abdominal infectious complications, defined as organ/space infections (OSIs). Secondary outcomes were rates of wound infections (hereafter referred to as superficial SSIs), POPF and bacteraemia, duration of hospital stay, and bile culture results. OSIs and superficial SSIs were classified according to the Centers for Disease Control and Prevention definition (*Appendix S2*)^16^. POPF was classified according to International Study Group of Pancreatic Surgery definitions^17^. Only clinically relevant POPF (grade B and C) was considered in the analyses. Bacteraemia was defined by the presence of a positive blood culture. Comparisons were made between patients with perioperative prophylaxis (either perioperative or for 24 h) and those with prolonged antibiotic prophylaxis (longer than 24 h). Additionally, subset analyses were undertaken for patients with preoperative biliary drainage.

### Statistical analysis

Quantitative analyses were performed using Review Manager (RevMan version 5.3, Copenhagen: The Nordic Cochrane Centre, Cochrane Collaboration). The *I*^2^ statistic was used to assess heterogeneity between studies. An *I*^2^ value of more than 50 per cent was considered to represent substantial heterogeneity. A Mantel–Haenszel fixed-effect model was used to calculate pooled effects, presented as ORs and 95 per cent confidence intervals.

## Results

### Study characteristics

**Fig. 1:**
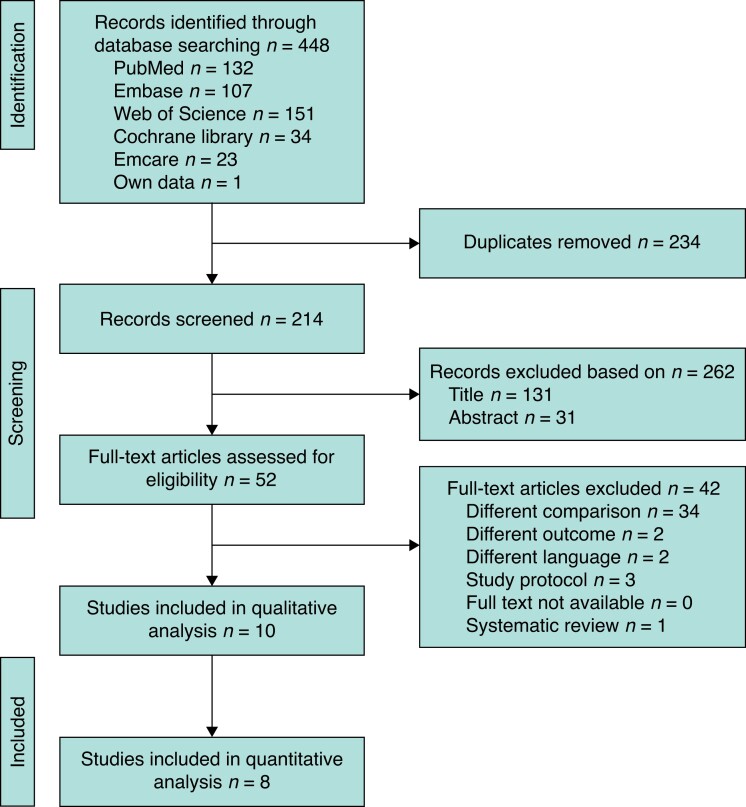
PRISMA diagram showing selection of articles for review

The literature search identified 448 studies. After removal of duplicates and detailed assessment of titles, abstracts, and full text, 10 studies were considered eligible (*Fig. 1*). Two observational studies^18,19^ were excluded from the quantitative analysis owing to a serious risk of bias as a result of a substantial baseline differences between patients receiving perioperative *versus* prolonged prophylaxis (patients without and with preoperative biliary drainage respectively) (*Tables 1* and *2*). Six observational studies and two RCTs reporting on 1170 patients were included (*Table 3*). Baseline characteristics, such as age, sex, smoking behaviour, and diabetes were similar between patients who received perioperative *versus* prolonged prophylaxis.

**Table 1:** Risk-of-bias assessment according to ROBINS-I tool for cohort studies

	Confounding	Selection of participants	Classification of intervention	Deviation from intended interventions	Missing data	Measurement of outcomes	Selection of reported results	Overall risk of bias
Sourrouille *et al.*^19^	Serious	Moderate	Low	Low	Low	Moderate	Low	Serious
Mohammed *et al*.^20^	Moderate	Moderate	Low	Moderate	Low	Low	Low	Moderate
Fathi *et al*.^21^	Low	Moderate	Low	Low	Low	Moderate	Low	Moderate
Degrandi *et al.*^22^	Low	Moderate	Low	Moderate	Low	Moderate	Low	Moderate
Sánchez Acedo *et al.*^18^	Serious	Moderate	Low	Low	Low	Low	Moderate	Serious
Petit *et al.*^23^	Moderate	Moderate	Low	Low	Moderate	Moderate	Low	Moderate
Fromentin *et al.*^24^	Moderate	Moderate	Moderate	Low	Low	Low	Low	Moderate
Droogh *et al.*^25^	Low	Moderate	Low	Low	Moderate	Low	Low	Moderate

ROBINS-I, Risk Of Bias In Non-randomized Studies—of Interventions

**Table 2:** Risk-of-bias assessment according to the Cochrane tool for randomised studies

	Randomization	Deviation from intended interventions	Missing data	Measurement of outcomes	Selection of reported result	Overall risk of bias
Okamura *et al*.^26^	Low	Low	Low	Low	Moderate	Moderate
Yamamoto *et al*.^27^	Low	Low	Low	Low	Low	Low

**Table 3:** Study characteristics

	Study design	Country	Inclusion interval	Inclusion criteria	Total sample size	Preoperative biliary drainage (%)	
						Perioperative prophylaxis	Prolonged prophylaxis
Sourrouille *et al.*^19^*	Prospective	France	2004–2009	Pancreatoduodenectomy	175	0	100
Mohammed *et al.*^20^	Retrospective	USA	2005–2011	Pancreatoduodenectomy	197	48	67
Fathi *et al.*^21^	Retrospective with PSM	USA	2006–2001	Pancreatoduodenectomy	74	59	78
Okamura *et al.*^26^	RCT	Japan	2008–2013	HPB surgery with biliary reconstruction†	38	100	100
Yamamoto *et al.*^27^	RCT	Japan	2012–2016	Pancreatoduodenectomy with preoperative biliary drainage‡	82	100	100
Degrandi *et al.*^22^	Retrospective	France	2008–2017	Pancreatoduodenectomy with preoperative biliary drainage	122	100	100
Sánchez Acedo *et al.*^18^*	Retrospective	Spain	2015–2018	Pancreatoduodenectomy	90	0	100
Petit *et al.*^23^	Retrospective	France	2007–2018	Major pancreatic surgery (77% pancreatoduodenectomy)	149	18	55
Fromentin *et al.*^24^	Retrospective	France	2010–2016	Pancreatoduodenectomy	146	100	100
Droogh *et al.*^25^	Retrospective	Netherlands	2016–2019	Pancreatoduodenectomy	362	56	46

Only included in qualitative analysis. †Only patients who underwent pancreatoduodenectomy included for analysis in this review. ‡Patients with cholangitis were excluded. PSM, propensity score matching.

The percentage of patients who underwent preoperative biliary drainage differed between the studies (*Table 3*). Four studies^20,21,23,25^ (782 patients) included all patients undergoing pancreatoduodenectomy. Preoperative biliary drainage was performed in 45.3 per cent of patients receiving perioperative prophylaxis and in 62.7 per cent of those receiving prolonged prophylaxis. Five studies^22,24–27^ (577 patients) reported the results for patients who underwent biliary drainage before pancreatoduodenectomy separately.

### Choice of antibiotic prophylaxis

Type of perioperative and prolonged antibiotics differed between the studies (*Table 4*). Perioperative antibiotic prophylaxis consisted of a second- or third-generation cephalosporin in seven studies. In eight studies, patients received a different antibiotic agent as prolonged prophylaxis compared with the perioperative antibiotic agent. The different antibiotic agent for the prolonged prophylaxis was based on individually obtained bile cultures (3 studies), bile cultures analysed in a former cohort of patients (4), or surgeon’s preference (1). The indication for prolonged antibiotic prophylaxis in the observational studies was based on positive bile cultures (4), perioperative observations (1), year of surgery (1) or was centre-specific (2). The duration of prolonged antibiotic prophylaxis was either 2 days (1), 5 days (4), or 10 days (2), or based on surgeon’s preference (1).

**Table 4:** Study antibiotics

	Sample size			Type of antibiotics		Duration of antibiotics	
	Perioperative	Prolonged	Indication for prolonged prophylaxis	Perioperative	Prolonged	Perioperative	Prolonged
Sourrouille *et al.*^19^*	76	99	Positive bile culture	Cefoxitin	Gentamicin, piperacillin, tazobactam (or ticarcillin + clavulanic acid)	Perioperative	5 days (2 days for negative IOBC)
Mohammed *et al.*^20^	128	69	Positive bile culture	Carbapenem	Tailored (IOBC)	24 h	10 days (3 days for negative IOBC)
Fathi *et al.*^21^	37	37	Positive bile culture	Third-generation cephalosporin + piperacillin, tazobactam + fluconazole	Tailored (IOBC)	24 h	10 days (3 days for negative IOBC)
Okamura *et al.*^26^	19	19	Randomization	Cefmetazole	Tailored (preoperative bile culture)	Perioperative	48 h
Yamamoto *et al.*^27^	40	42	Randomization	Cefozopran	Cefozopran	24 h	5 days
Degrandi *et al.*^22^	53	69	Year of surgery	Cefazolin	Piperacillin + tazobactam	Perioperative	5 days
Sánchez Acedo *et al*.^18^*	39	51	Preoperative biliary drainage	Cefoxitin	Piperacillin + tazobactam	Perioperative	5 days
Petit *et al.*^23^	107	42	Perioperative signs of infection	Cefuroxime	Surgeon’s preference	Perioperative	Not protocolized
Fromentin *et al.*^24^	65	81	Centre-specific	Cefazolin, cefoxitin or cefamandole	Piperacillin + tazobactam, in one centre additional dose of gentamicin	Perioperative	5 days
Droogh *et al.*^25^	219	143	Centre-specific	Cefazoline + metronidazole	Cefuroxime + metronidazole	Perioperative	5 days

Only included in qualitative analysis. IOBC, intraoperative bile culture.

### Organ/space infections

**Fig. 2:**
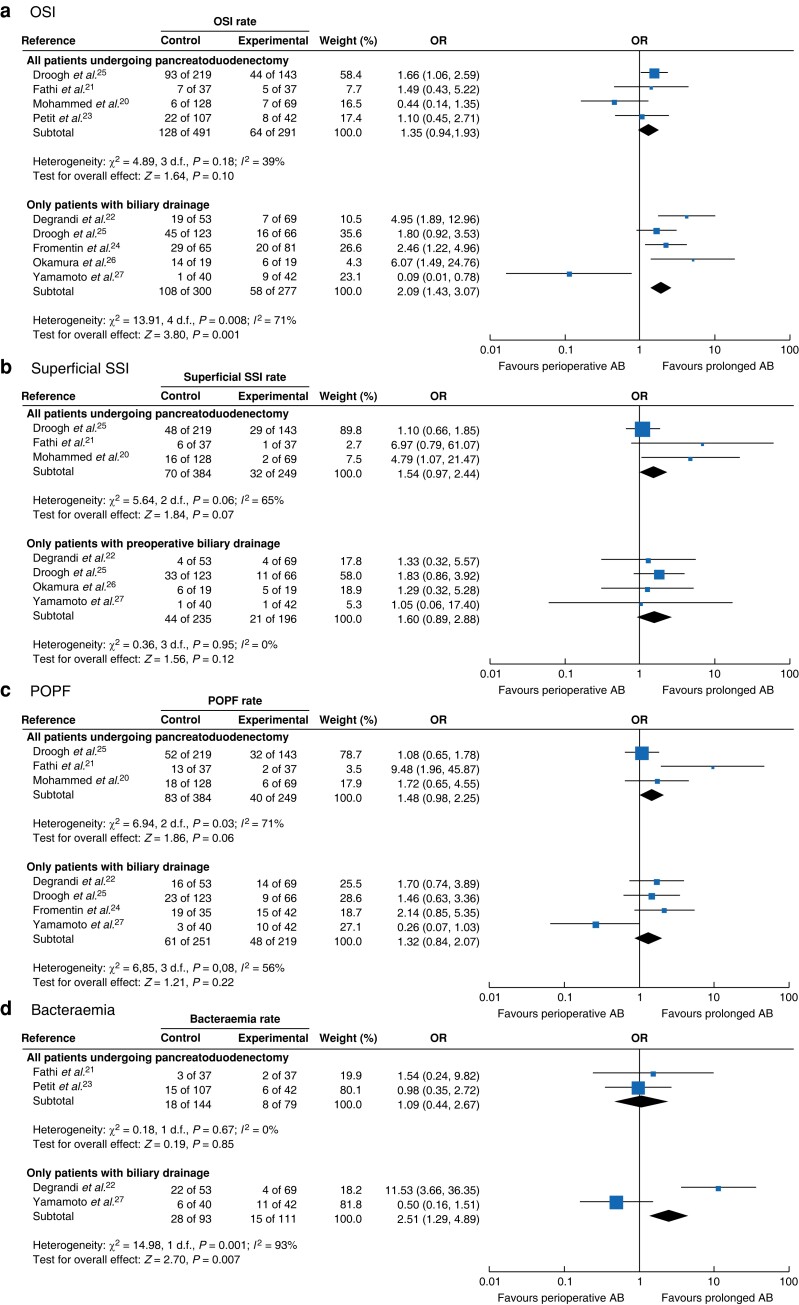
Forest plots showing occurrence of organ/space infection, superficial surgical-site infection, postoperative pancreatic fistula, and bacteraemia in patients with perioperative *versus* prolonged prophylaxis **a** Organ/space infection (OSI), **b** superficial surgical-site infection (SSI), **c** postoperative pancreatic fistula (POPF), and **c** bacteraemia. For each complication, results are shown for all patients undergoing pancreatoduodenectomy and only in those who underwent preoperative biliary drainage. A Mantel–Haenszel fixed-effect model was used for meta-analysis. ORs are shown with 95% confidence intervals. AB, antibiotics.

Eight studies (1170 patients) were included in the quantitative analysis of OSIs (*Figs 2* and *S2*). Four studies (782 patients) included all patients undergoing pancreatoduodenectomy, and showed comparable OSI rates in patients who had perioperative *versus* prolonged prophylaxis (pooled OR 1.35, 95 per cent c.i. 0.94 to 1.93; *I*^2^ = 39 per cent). Five studies (577 patients) included patients with biliary drainage performed before pancreatoduodenectomy, and observed a lower OSI rate in patients receiving prolonged antibiotic prophylaxis (pooled OR 2.09, 1.43 to 3.07; *I*^2^ = 71 per cent). Owing to substantial heterogeneity of the studies, an additional analysis using a Mantel–Haenszel random-effects model was carried out for OSIs in patients with biliary drainage, which demonstrated an OR of 2.19 (0.94 to 5.07) (*Fig. S1*).

### Superficial surgical-site infections

Six studies (875 patients) were included in the quantitative analysis of superficial SSIs (*Figs 2* and *S2*). Three studies (633 patients) included all patients undergoing pancreatoduodenectomy, and did not show a difference in superficial SSI rates between patients with perioperative *versus* prolonged prophylaxis (pooled OR 1.54, 95 per cent c.i. 0.97 to 2.44; *I*^2^ = 65 per cent). Four studies (431 patients) included only patients with preoperative biliary drainage, and did not show a difference in superficial SSI rates in patients with perioperative *versus* prolonged prophylaxis (pooled OR 1.60, 0.89 to 2.88; *I*^2^ = 0 per cent).

### Postoperative pancreatic fistula

Six studies (914 patients) were included in the quantitative analysis for POPF (*Figs 2* and *S2*). Three studies (633 patients) included all patients undergoing pancreatoduodenectomy, and observed comparable POPF rates in patients who received perioperative *versus* prolonged prophylaxis (pooled OR 1.48, 95 per cent c.i. 0.98 to 2.25; *I*^2^ = 71 per cent). Four studies (470 patients) included only patients who had preoperative biliary drainage, and showed similar POPF rates in patients with perioperative *versus* prolonged prophylaxis (pooled OR 1.32, 0.84 to 2.07; *I*^2^ = 56 per cent).

### Bacteraemia

Four studies (427 patients) were included in the quantitative analysis of bacteraemia (*Figs 2* and *S2*). Two studies (223 patients) included all patients undergoing pancreatoduodenectomy, and did not show a difference in bacteraemia rates between patients who had perioperative *versus* prolonged prophylaxis (pooled OR 1.09, 95 per cent c.i. 0.44 to 2.67; *I*^2^ = 0 per cent). Two studies (204 patients) included only patients who had preoperative biliary drainage and reported more bacteraemia in patients with perioperative prophylaxis only (pooled OR 2.51, 1.29 to 4.89; *I*^2^ = 93 per cent).

### Duration of hospital stay

Four studies (475 patients) were included in the quantitative analysis of hospital stay (*Fig. S3*). Two studies (271 patients) included all patients undergoing pancreatoduodenectomy, and did not report a difference in duration of hospital stay for patients who received perioperative *versus* prolonged prophylaxis (pooled mean difference −0.09 (95 per cent c.i. −0.38 to 0.20) days; *I*^2^ = 0 per cent). Two studies (204 patients) analysed duration of hospital stay in patients who had preoperative biliary drainage, and reported only median (i.q.r.) values. Yamamoto *et al.*^27^ documented a median duration of hospital stay of 10 (8–33 days) days for patients who received perioperative prophylaxis and 15 (8–44) days for those with prolonged prophylaxis (*P* = 0.018). Degrandi *et al.*^22^ reported 17 (13–27) and 13 (10–14) days respectively (*P* < 0.001).

### Microbiology

Six studies examined the results of culture of bile samples obtained during surgery; the cultures were predominantly polymicrobial (range 54–69 per cent)^18–22,26^. The most frequently cultured microorganisms were *Enterococci* (range 14–63 per cent), *Enterobacter* (range 22–30 per cent), and *Klebsiella* (range 18–39 per cent) species. Three studies^21,22,26^ compared microbiological profiles of bile and abdominal drain cultures, and reported a concordance of 12–39 per cent. Degrandi *et al.*^22^ showed more extensive resistance rates for microorganisms cultured from bile in patients receiving only perioperative antibiotics (first-generation cephalosporin) compared with prolonged antibiotics (piperacillin with tazobactam): 64 *versus* 14 per cent. Three studies^23,26,27^ reported on resistance patterns of postoperative cultures from SSIs, and reported similar resistance rates between patients with perioperative *versus* prolonged prophylaxis, including the proportion of multidrug-resistant microorganisms.

## Discussion

This systematic review and meta-analysis did not demonstrate a difference in OSI between patients receiving perioperative *versus* prolonged antibiotics (OR 1.35, 95 per cent c.i. 0.94 to 1.93). However, there was a lower proportion of OSIs in patients who had preoperative biliary drainage receiving prolonged antibiotic prophylaxis after pancreatoduodenectomy (OR 2.09, 1.43 to 3.07). The rate of superficial SSIs and POPF was comparable between patients receiving perioperative *versus* prolonged antibiotic prophylaxis, also after stratification for preoperative biliary drainage. Moreover, antibiotic resistance rates were comparable between patients receiving perioperative or prolonged prophylaxis.

SSIs and POPF occur frequently after pancreatoduodenectomy, and previous research^2,4,28^ related these complications to contaminated bile. In line with these studies, the present overview showed an association between positive bile cultures and the occurrence of OSIs and bacteraemia. However, an association between contaminated bile and pancreatic fistula was not demonstrated. Optimization of postoperative antibiotic prophylaxis potentially decreases the rate of OSIs and bacteraemia, which might lead to a shorter hospital admission and faster time to functional recovery; this was reported by only one study^22^ in this review. However, duration of hospital admission was not extended by the prolonged administration of antibiotics. This review accentuated the varying administration and duration of antibiotic prophylaxis between institutes. Current guidelines lack clear recommendations regarding type and duration of antibiotic prophylaxis; as with the current evidence, the optimal antibiotic prophylaxis for patients with a high risk of contaminated bile remains undetermined^9^.

Perioperative antibiotic prophylaxis is used widely to prevent SSIs, whereas prolonged antibiotic prophylaxis could be used as pre-emptive treatment to prevent OSIs in surgical procedures with a risk of perioperative contamination. A recent meta-analysis^8^ evaluated the effect of targeted antibiotic prophylaxis based on bile cultures obtained from a former cohort of patients, and reported a 21 per cent decrease in SSIs. However, not only type but also duration of the antibiotic regimens differed substantially between the included studies. Standard use of prolonged antibiotic prophylaxis in patients with contaminated bile could replace individually tailored antibiotic prophylaxis to reduce abdominal infectious complications, as supported by the results of this review. Preoperative biliary instrumentation and the presence of an ampullary malignancy are highly associated with contaminated bile, as approximately 95 per cent of these patients have positive bile cultures^2–4,29^. As use of preoperative biliary drainage is likely to increase owing to neoadjuvant therapies, antibiotic prophylaxis should be optimized for these patients. Hence, prolonged antibiotic prophylaxis should be considered for patients with a high risk of contaminated bile to reduce OSIs.

Various other interventions have been investigated to prevent SSIs. Recently, a multicentre RCT^30^ including 13 301 patients demonstrated a 3 per cent lower SSI rate after routine change of gloves before wound closure in abdominal surgery with contamination of the abdominal space. Wound management devices have also been suggested to reduce SSIs in abdominal and biliary surgery^31^. However, an RCT^32^ of 212 patients undergoing pancreatoduodenectomy did not demonstrate an effect of an intraoperative wound protector device on the superficial SSI rate. Additionally, a meta-analysis^33^ including 4 studies reporting on 309 patients did not demonstrate a benefit of negative pressure wound therapy on superficial SSIs. Fewer interventions to prevent OSIs have been evaluated. A small RCT^34^ including 40 patients evaluated bile duct clamping during pancreatoduodenectomy, and did not demonstrate a difference in OSI between patients with or without bile duct clamping (4 *versus* 11 OSIs; *P* = 0.23). Antibiotic *versus* saline irrigation during pancreatoduodenectomy was evaluated in a RCT^35^ including 190 patients, which reported comparable superficial SSI and OSI rates. Overall, preventive interventions, aside from adequate antibiotic prophylaxis, have not been confirmed to substantially reduce OSIs after pancreatoduodenectomy.

In most of the studies included in this review, type of prolonged antibiotic prophylaxis was based on bile cultures, which were either patient-specific (based on cultures obtained during surgery which are generally available after 3–5 postoperative days), or standardized based on bile culture results of a former cohort of patients. The microbiological profile of bile cultures was commonly polymicrobial, and predominantly showed *Enterococcus*, *Enterobacter*, and *Klebsiella* species. *Enterococcus* and *Enterobacter* species are intrinsically resistant to antibiotic agents frequently used as antibiotic prophylaxis (for example first- to third-generation cephalosporins and nitroimidazole derivates), whereas *Klebsiella* species could develop acquired resistance to cephalosporins^36–38^. Although *Enterococcus* species were often present in (mainly polymicrobial) bile and abdominal drain fluid cultures, *Enterococci* are considered low-virulence microorganisms^36,39^. Previous studies^40,41^ supported a sufficient effect of cephalosporins combined with metronidazole as prophylaxis with regard to SSIs. In the present review, there was a comparable prevalence of the abovementioned bacteria in patients receiving perioperative *versus* prolonged prophylaxis. Despite the presence of these bacteria, the overall rate of abdominal infectious complications was lower in patients receiving prolonged prophylaxis. It is plausible that adequate antibiotic prophylaxis does not necessarily require coverage of *Enterococcus* species.

One of the main drawbacks of prolonged antibiotic prophylaxis is the potential acquisition of antibiotic resistance by selective antibiotic pressure at both the individual and population level^42^. Many hospitals have antibiotic stewardship programmes to tackle the threat of increasing antibiotic resistance by restrictive and appropriate antibiotic use^43^. The benefit of prolonged antibiotic prophylaxis should be clearly established before widespread implementation. Increasing antibiotic resistance in turn might limit the arsenal of antibiotics suited for prophylaxis, particularly in the event of two different standards regarding type of antibiotic prophylaxis for patients with and without contaminated bile. Recently, two Dutch studies^3,29^ showed low resistance rates of microorganisms cultured from bile obtained during surgery towards perioperative prophylaxis (cefazolin and metronidazole). In three of the studies included in this review, susceptibility patterns of bacteria cultured from bile and abdominal fluid also demonstrated low acquired resistance rates to the antibiotics used for perioperative or prolonged prophylaxis^23,26,27^. Nevertheless, resistance patterns vary substantially by region and should therefore be monitored when administering prolonged antibiotic prophylaxis. Long-term outcomes regarding antimicrobial resistance and the effect of prolonged antibiotics on the intestinal microbiome should be evaluated when administering prolonged prophylaxis, particularly with regard to pancreatic cancer outcomes^44^.

The heterogeneity of the included studies in terms of patient selection, and type and duration of antibiotic prophylaxis is one of the main limitations of this review. In addition, data on the clinical impact of fewer abdominal infections in terms of reinterventions and readmissions was limited. Furthermore, six of the included studies were observational and the only two RCTs reported conflicting results. The negative effect of prolonged antibiotics on OSIs reported by Yamamoto *et al.*^27^ might be explained by the high POPF rate in the prolonged prophylaxis group, and could be affected by the small sample size. Nevertheless, antibiotic prophylaxis was administered for 5 days in four of five studies evaluating patients with contaminated bile, and a substantial difference was measured in primary outcome.

Prolonged antibiotic prophylaxis seems to lower the rate of OSIs in patients undergoing pancreatoduodenectomy with contaminated bile, without increasing short-term bacterial resistance rates. The promising effect of prolonged antibiotic prophylaxis for patients with preoperative biliary drainage warrants evaluation in an adequately powered RCT.

## Supplementary Material

znad213_Supplementary_Data

## Data Availability

All data analysed during this study are included in this article (and its *supplementary material*) as references to published articles, or are available from the corresponding authors on reasonable request.
