# Supplementary material for: Prolonged antibiotic prophylaxis after pancreatoduodenectomy: systematic review and meta-analysis
Source: Br J Surg. 2023 Jul 13;110(11):1458–66. doi: 10.1093/bjs/znad213 (PMC10564402; doi:10.1093/bjs/znad213)

**Supplementary**

**Prolonged antibiotic prophylaxis after pancreatoduodenectomy: a systematic review and meta-analysis**

D.H.M. Droogh^1^, J.V. Groen^1^, M.G.J. de Boer^2^, J. van Prehn^3^, H. Putter^4^, B.A. Bonsing^1^, C.H.J. van Eijck^5^, A.L. Vahrmeijer^1^, H.C. van Santvoort^6^, B. Groot Koerkamp^5^, J.S.D. Mieog^1^

*^1^ Department of Surgery, Leiden University Medical Center, Leiden, The Netherlands
^2^ Departments of Infectious Diseases & Clinical Epidemiology, Leiden University Medical Center, Leiden, The Netherlands*

*^3^ Department of Medical Microbiology, Leiden University Medical Center, Leiden, The Netherlands*

*^4^ Department of Biomedical Data Sciences, Leiden University Medical Center, Leiden, The Netherlands*

*^5^ Department of Surgery, Erasmus MC Cancer Institute, Rotterdam, The Netherlands*

*^6^ Department of Surgery, Regional Academic Cancer Center Utrecht (RAKU), Utrecht, The Netherlands*

**Corresponding author and reprint requests:**

D.H.M. Droogh, MD

Department of Surgery, Leiden University Medical Center

Albinusdreef 2, 2333 ZA Leiden, The Netherlands

Tel: +31 71 526 1334

Mail: [D.H.M.Droogh@lumc.nl](mailto:D.H.M.Droogh@lumc.nl)

**Supplementary Materials – Index**

| **Supplementary Methods** |  |
| --- | --- |
| Appendix S1. Literature search for PubMed | *pag. 3* |
| Appendix S2. CDC definition for superficial SSIs and OSIs | *pag. 5* |
| **Supplementary Figures and Tables** |  |
| Figure S1. OSI in patients with perioperative versus prolonged prophylaxis, calculated using the Mantel-Haenszel random-effects model. | *pag. 6* |
| Figures S2. Funnel plot OSI (A), superficial SSI (B), POPF (C), bacteraemia (D) | *pag. 7* |
| Figures S3. Length of hospital stay in patients with perioperative versus prolonged prophylaxis | *pag. 8* |
|  |  |

**Supplementary Methods**

**Appendix S1. Literature search for PubMed**

(("Antibiotic Prophylaxis"[Mesh] OR "Antibiotic Prophylaxis"[tw] OR "Antibiotic Premedication"[tw] OR "antimicrobial prophylaxis"[tw] OR "antibiotic therapy"[tw] OR "antibiotic treatment"[tw] OR "antimicrobial therapy"[tw] OR "antimicrobial treatment"[tw] OR (("Anti-Bacterial Agents"[Mesh] OR "Anti-Bacterial Agents"[Pharmacological Action] OR antibiotic*[tw] OR anti-biotic*[tw] OR antibacterial*[tw] OR anti-bacterial*[tw] OR antimicrobial*[tw] OR anti-microbial*[tw] OR "(2E, 2E)-4,4-trisulfanediylbis(but-2-enoic acid)"[tw] OR "1-(4-methoxyphenyl)-3-acetamido-4-methoxyazetidin-2-one"[tw] OR "2,2-dimethylpenam sulfone"[tw] OR "2,4-diacetylphloroglucinol"[tw] OR "2-(ethoxycarbonyl)-6,6-dihydropenicillanic acid"[tw] OR "2-deoxystreptamine"[tw] OR "3-(2'-benzyl-3'-mercaptopropanoyl)-4-carboxy-5,5-dimethylthiazolidine"[tw] OR "3-carboxyphenyl phenylacetamidomethylphosphonate"[tw] OR "3-carboxyphenyl((N-((4-iodophenyl)acetyl)amino)methyl)phosphonate"[tw] OR "3-hydroxy-2-methyl-4H-pyran-4-thione"[tw] OR "4-(3-amido-4-phenyl-2-azetidinonyl)-1-acetophenone"[tw] OR "4-(3-amido-4-phenyl-2-azetidinonyl)-1-phenylacetic acid"[tw] OR "4-(3-amino-2-oxoazetidinonyl-1)methylbenzoic acid"[tw] OR "4-(3-amino-2-oxoazetidinonyl-1)methylcyclohexanecarboxylic acid"[tw] OR "6,6-bis(hydroxylmethyl)penicillanate"[tw] OR "6-((4-nitrobenzyloxycarbonyl)methylsulfonamido)penicillanic acid sulfone"[tw] OR "6-((anilinocarbonyl)methylsulfonamido)penicillanic acid sulfone"[tw] OR "6-((carboxy)methylsulfonamido)penicillanic acid sulfone"[tw] OR "6-(1-hydroxy)benzylpenicillanic acid S, S-dioxide"[tw] OR "6-(methyoxymethylene)penicillanic acid"[tw] OR "6-acetylmethylenepenicillanic acid"[tw] OR "6-beta-(trifluoromethanesulfonyl)amido-penicillanic acid sulfone"[tw] OR "6-chloro-2-chloromethyl-2-methylpenam-3-carboxylic acid 1,1-dioxide"[tw] OR "6-iodopenicillanic acid"[tw] OR "6-methyl-2-aminopyridine palladium dichloride"[tw] OR "6-sulfoaminopenicillanic acid"[tw] OR "9-N-isobutylaminodeoxyclavulanate"[tw] OR "9-O-methylclavulanate"[tw] OR "Acedapsone"[tw] OR "Acetic Acid"[tw] OR "aconiazide"[tw] OR "actinonin"[tw] OR "actinorhodin"[tw] OR "Alamethicin"[tw] OR "albomycin"[tw] OR "AM 113"[tw] OR "AM 114"[tw] OR "AM 115"[tw] OR "Amdinocillin"[tw] OR "Amdinocillin Pivoxil"[tw] OR "amifloxacin"[tw] OR "Amikacin"[tw] OR "Aminosalicylic Acid"[tw] OR "Amoxicillin"[tw] OR "Amoxicillin-Potassium Clavulanate Combination"[tw] OR "amphomycin"[tw] OR "Amphotericin B"[tw] OR "Ampicillin"[tw] OR "amprenavir"[tw] OR "angustmycin A"[tw] OR "Anisomycin"[tw] OR "antibiotic 1233A"[tw] OR "Antimycin A"[tw] OR "antofloxacin"[tw] OR "apramycin"[tw] OR "Arsphenamine"[tw] OR "Aurodox"[tw] OR "AVE 1330A"[tw] OR "avibactam"[tw] OR "avibactam, ceftazidime drug combination"[tw] OR "avilamycin"[tw] OR "Azithromycin"[tw] OR "Azlocillin"[tw] OR "Aztreonam"[tw] OR "bacampicillin"[tw] OR "Bacitracin"[tw] OR "bacitracin zinc, neomycin sulfate, polymyxin B, drug combination"[tw] OR "bacitracin, cysteine, glycine, neomycin, threonine drug combination"[tw] OR "Bacteriocins"[tw] OR "balofloxacin"[tw] OR "Bambermycins"[tw] OR "bedaquiline"[tw] OR "bekanamycin"[tw] OR "benzathine benzylpenicillin, procaine benzylpenicillin, drug combination"[tw] OR "benzathine cloxacillin"[tw] OR "benzo(b)thiophene-2-boronic acid"[tw] OR "benzo(b)thiophene-2-ylboronic acid"[tw] OR "benzo(d)thiazole-2-carbanilide"[tw] OR "benzofuroquinolinium"[tw] OR "berythromycin"[tw] OR "besifloxacin"[tw] OR "beta-lactamase-inhibitor protein, Streptomyces"[tw] OR "beta-Lactams"[tw] OR beta-Lactam*[tw] OR "betalactams"[tw] OR betalactam*[tw] OR "bialaphos"[tw] OR "bicozamycin"[tw] OR "BL-P 2013"[tw] OR "BL-P 2090"[tw] OR "blasticidin S"[tw] OR "BLI-489"[tw] OR "BM 212"[tw] OR "Bongkrekic Acid"[tw] OR "Brefeldin A"[tw] OR "broadcillin"[tw] OR "brobactam"[tw] OR "Butirosin Sulfate"[tw] OR "C6-(N1-methyl-1,2,3-trazolylmethylene)penem"[tw] OR "cactinomycin"[tw] OR "Calcimycin"[tw] OR "Candicidin"[tw] OR "Capreomycin"[tw] OR "Carbenicillin"[tw] OR "carbenicillin indanyl"[tw] OR "Carfecillin"[tw] OR "catechol functionalized-chitosan"[tw] OR "Cefaclor"[tw] OR "Cefadroxil"[tw] OR "Cefamandole"[tw] OR "cefamandole nafate"[tw] OR "Cefatrizine"[tw] OR "cefazedone"[tw] OR "Cefazolin"[tw] OR "Cefdinir"[tw] OR "cefditoren"[tw] OR "cefditoren pivoxil"[tw] OR "Cefepime"[tw] OR "cefetamet"[tw] OR "cefetamet pivoxyl"[tw] OR "Cefixime"[tw] OR "Cefmenoxime"[tw] OR "Cefmetazole"[tw] OR "cefminox"[tw] OR "cefodizime"[tw] OR "Cefonicid"[tw] OR "Cefoperazone"[tw] OR "ceforanide"[tw] OR "cefoselis"[tw] OR "Cefotaxime"[tw] OR "Cefotetan"[tw] OR "Cefotiam"[tw] OR "cefotiam hexetil"[tw] OR "Cefoxitin"[tw] OR "cefpimizole"[tw] OR "cefpiramide"[tw] OR "cefpirome"[tw] OR "cefpodoxime"[tw] OR "cefpodoxime proxetil"[tw] OR "cefprozil"[tw] OR "Cefsulodin"[tw] OR "ceftaroline fosamil"[tw] OR "Ceftazidime"[tw] OR "cefteram pivoxil"[tw] OR "ceftezole"[tw] OR "Ceftibuten"[tw] OR "ceftiofur"[tw] OR "Ceftizoxime"[tw] OR "ceftobiprole"[tw] OR "ceftobiprole medocaril"[tw] OR "ceftolozane"[tw] OR "ceftolozane, tazobactam drug combination"[tw] OR "Ceftriaxone"[tw] OR "Cefuroxime"[tw] OR "cefuroxime axetil"[tw] OR "Cephacetrile"[tw] OR "Cephalexin"[tw] OR "Cephaloglycin"[tw] OR "Cephaloridine"[tw] OR "Cephalosporins"[tw] OR "Cephalosporin"[tw] OR Cephalosporin*[tw] OR "Cephalothin"[tw] OR "cephamycin C"[tw] OR "Cephamycins"[tw] OR "Cephapirin"[tw] OR "Cephradine"[tw] OR "cethromycin"[tw] OR "CH 1240"[tw] OR "CH 2140"[tw] OR "chelerythrine"[tw] OR "Chloramphenicol"[tw] OR "chloramphenicol succinate"[tw] OR "chlorotetracycline, penicillin G, sulfamethazine drug combination"[tw] OR "chloroxine"[tw] OR "Chlortetracycline"[tw] OR "Cilastatin, Imipenem Drug Combination"[tw] OR "Ciprofloxacin"[tw] OR "ciprofloxacin, hydrocortisone drug combination"[tw] OR "Citrinin"[tw] OR "Clarithromycin"[tw] OR "Clavulanic Acid"[tw] OR "Clavulanic Acids"[tw] OR "clinafloxacin"[tw] OR "Clindamycin"[tw] OR "clindamycin palmitate"[tw] OR "clindamycin phosphate"[tw] OR "Clofazimine"[tw] OR "Cloxacillin"[tw] OR "Colistin"[tw] OR "CS 834"[tw] OR "Cyclacillin"[tw] OR "Cycloserine"[tw] OR "Dactinomycin"[tw] OR "dalbavancin"[tw] OR "dalfopristin"[tw] OR "Dapsone"[tw] OR "Daptomycin"[tw] OR "decamethoxine"[tw] OR "delpazolid"[tw] OR "Demeclocycline"[tw] OR "desoxyfructo-serotonin"[tw] OR "dexamethasone, neomycin, polymyxin B drug combination"[tw] OR "Diarylquinolines"[tw] OR "Dibekacin"[tw] OR "Dicloxacillin"[tw] OR "Dihydrostreptomycin Sulfate"[tw] OR "Diketopiperazines"[tw] OR "dirithromycin"[tw] OR "Distamycins"[tw] OR "diucifon"[tw] OR "Doripenem"[tw] OR "dotriacolide"[tw] OR "Doxycycline"[tw] OR "dynemicin A"[tw] OR "Echinomycin"[tw] OR "Edeine"[tw] OR "efrotomycin"[tw] OR "emiglitate"[tw] OR "Enoxacin"[tw] OR "Enrofloxacin"[tw] OR "Enviomycin"[tw] OR "epicillin"[tw] OR "Ertapenem"[tw] OR "Erythromycin"[tw] OR "Erythromycin Estolate"[tw] OR "Erythromycin Ethylsuccinate"[tw] OR "erythromycin lactobionate"[tw] OR "erythromycin stearate"[tw] OR "essential 303 forte"[tw] OR "etamycin"[tw] OR "Ethambutol"[tw] OR "Ethionamide"[tw] OR "etimicin"[tw] OR "Fidaxomicin"[tw] OR "Filipin"[tw] OR "finafloxacin"[tw] OR "FK 565"[tw] OR "florfenicol"[tw] OR "Floxacillin"[tw] OR "Fluoroquinolones"[tw] OR "forphenicinol"[tw] OR "Fosfomycin"[tw] OR "fosmidomycin"[tw] OR "Framycetin"[tw] OR "fumagillin"[tw] OR "fusafungin"[tw] OR "Fusidic Acid"[tw] OR "gamithromycin"[tw] OR "garenoxacin"[tw] OR "Gatifloxacin"[tw] OR "GE 2270 A"[tw] OR "Gemifloxacin"[tw] OR "Gentamicins"[tw] OR "geranyl cinnamate"[tw] OR "Gramicidin"[tw] OR "grepafloxacin"[tw] OR "herbimycin"[tw] OR "hydrocortisone, neomycin, polymyxin B drug combination"[tw] OR "Hygromycin B"[tw] OR "IIIM-MCD-211"[tw] OR "IIS-PAA"[tw] OR "Imipenem"[tw] OR "immunomycin"[tw] OR "isatoic anhydride"[tw] OR "isepamicin"[tw] OR "Isoniazid"[tw] OR "isoniazid, pyrazinamide, rifampin drug combination"[tw] OR "izumenolide"[tw] OR "Josamycin"[tw] OR "KA-107"[tw] OR "Kanamycin"[tw] OR "Kitasamycin"[tw] OR "KRM 1648"[tw] OR "KT 5720"[tw] OR "L 159906"[tw] OR "lactacystin"[tw] OR "Lactams"[tw] OR "lacticin 481"[tw] OR "lactoferricin B"[tw] OR "Lasalocid"[tw] OR "lefamulin"[tw] OR "Leucomycins"[tw] OR "Levofloxacin"[tw] OR "Lincomycin"[tw] OR "Lincosamides"[tw] OR "Linezolid"[tw] OR "LN 1-255"[tw] OR "lomefloxacin"[tw] OR "loracarbef"[tw] OR "LPC-058"[tw] OR "Lucensomycin"[tw] OR "lydiamycin A"[tw] OR "Lymecycline"[tw] OR "lysobactin"[tw] OR "macozinone"[tw] OR "maduramicin"[tw] OR "Mafenide"[tw] OR "malacidins"[tw] OR "maltotetraose"[tw] OR "manoalide"[tw] OR "manumycin"[tw] OR "marbofloxacin"[tw] OR "marcellomycin"[tw] OR "meclocycline"[tw] OR "Mepartricin"[tw] OR "Meropenem"[tw] OR "Methacycline"[tw] OR "methampicillin"[tw] OR "methenamine hippurate"[tw] OR "methenamine mandelate"[tw] OR "Methicillin"[tw] OR "methylene-6-(3-formylallylidene)penicillanate pivalate"[tw] OR "Metronidazole"[tw] OR "mevastatin"[tw] OR "Mezlocillin"[tw] OR "micronomicin"[tw] OR "midecamycin"[tw] OR "Mikamycin"[tw] OR "Minocycline"[tw] OR "Miocamycin"[tw] OR "mirincamycin"[tw] OR "mizoribine"[tw] OR "MK 8712"[tw] OR "mocimycin"[tw] OR "Moxalactam"[tw] OR "Moxifloxacin"[tw] OR "muconomycin A"[tw] OR "Mupirocin"[tw] OR "muraymycin D1"[tw] OR "murepavadin"[tw] OR "Mycobacillin"[tw] OR "Mycophenolic Acid"[tw] OR "N-(2'-mercaptoethyl)-2-phenylacetamide"[tw] OR "N-halamine"[tw] OR "N-methyldeoxynojirimycin"[tw] OR "N-tosyloxy-3-phthalimido-4-methyl-2-azetidinone"[tw] OR "N-tosyloxy-4-phenyl-2-azetidinone"[tw] OR "nadifloxacin"[tw] OR "Nafcillin"[tw] OR "nafithromycin"[tw] OR "Nalidixic Acid"[tw] OR "narasin"[tw] OR "Natamycin"[tw] OR "Nebacetin"[tw] OR "Nebramycin"[tw] OR "nebularine"[tw] OR "Neomycin"[tw] OR "Netilmicin"[tw] OR "Netropsin"[tw] OR "Nigericin"[tw] OR "Nisin"[tw] OR "nisin Z"[tw] OR "Nitrofurantoin"[tw] OR "nojirimycin"[tw] OR "Norfloxacin"[tw] OR "norvancomycin"[tw] OR "Novobiocin"[tw] OR "Nystatin"[tw] OR "Ofloxacin"[tw] OR "Oleandomycin"[tw] OR "Oligomycins"[tw] OR "olivacins"[tw] OR "oritavancin"[tw] OR "ormetoprim, sulfadimethoxine drug combination"[tw] OR "Oxacillin"[tw] OR "oxetanocin"[tw] OR "Oxolinic Acid"[tw] OR "Oxytetracycline"[tw] OR "panipenem-betamipron"[tw] OR "Paromomycin"[tw] OR "pazufloxacin"[tw] OR "pediocin PA-1"[tw] OR "Pefloxacin"[tw] OR "Penicillanic Acid"[tw] OR "Penicillic Acid"[tw] OR "Penicillin G"[tw] OR "Penicillin G Benzathine"[tw] OR "Penicillin G Procaine"[tw] OR "Penicillin V"[tw] OR "Penicillin"[tw] OR "Penicillins"[tw] OR penicillin*[tw] OR "penimepicycline"[tw] OR "pexiganan"[tw] OR "phenethicillin"[tw] OR "phenylpropynal"[tw] OR "phosphoramidon"[tw] OR "piericidin A"[tw] OR "Pipemidic Acid"[tw] OR "Piperacillin"[tw] OR "Piperacillin, Tazobactam Drug Combination"[tw] OR "Pivampicillin"[tw] OR "pleuromutilin"[tw] OR "pluracidomycin"[tw] OR "Polymyxin B"[tw] OR "polymyxin B(1)"[tw] OR "Polymyxins"[tw] OR "polyoxorim"[tw] OR "PR 39"[tw] OR "Pristinamycin"[tw] OR "Prodigiosin"[tw] OR "propargyl bromide"[tw] OR "propicillin"[tw] OR "Prothionamide"[tw] OR "prulifloxacin"[tw] OR "Pyrazinamide"[tw] OR "pyrazofurin"[tw] OR "quinupristin"[tw] OR "quinupristin-dalfopristin"[tw] OR "radezolid"[tw] OR "ramoplanin"[tw] OR "relebactam"[tw] OR "retapamulin"[tw] OR "Ribostamycin"[tw] OR "Rifabutin"[tw] OR "rifamexil"[tw] OR "Rifampin"[tw] OR "Rifamycins"[tw] OR "rifapentine"[tw] OR "Rifaximin"[tw] OR "Ristocetin"[tw] OR "Ro 48-1220"[tw] OR "Ro 48-1256"[tw] OR "Ro 48-5545"[tw] OR "Ro 48-8724"[tw] OR "Rolitetracycline"[tw] OR "Roxarsone"[tw] OR "Roxithromycin"[tw] OR "Rutamycin"[tw] OR "saframycin A"[tw] OR "salinomycin"[tw] OR "sangivamycin"[tw] OR "SB 202742"[tw] OR "Sirolimus"[tw] OR "Sisomicin"[tw] OR "sitafloxacin"[tw] OR "SM-4300"[tw] OR "sodium thiosulfate"[tw] OR "solithromycin"[tw] OR "sparfloxacin"[tw] OR "Spectinomycin"[tw] OR "Spiramycin"[tw] OR "squalamine"[tw] OR "staphylococcin"[tw] OR "stigmatellin"[tw] OR "Streptogramin A"[tw] OR "Streptogramin Group A"[tw] OR "Streptogramin Group B"[tw] OR "Streptogramins"[tw] OR "streptolydigin"[tw] OR "Streptomycin"[tw] OR "Streptovaricin"[tw] OR "Sulbactam"[tw] OR "Sulbenicillin"[tw] OR "Sulfacetamide"[tw] OR "Sulfadiazine"[tw] OR "Sulfamerazine"[tw] OR "Sulfameter"[tw] OR "Sulfamethoxypyridazine"[tw] OR "Sulfanilamide"[tw] OR "sultamicillin"[tw] OR "suncillin"[tw] OR "SYN 1012"[tw] OR "Syn 2190"[tw] OR "syringomycin"[tw] OR "Talampicillin"[tw] OR "Tazobactam"[tw] OR "tedizolid"[tw] OR "tedizolid phosphate"[tw] OR "Teicoplanin"[tw] OR "telavancin"[tw] OR "telithromycin"[tw] OR "temafloxacin"[tw] OR "temocillin"[tw] OR "tetarimycin A"[tw] OR "tetracenomycin C"[tw] OR "Tetracycline"[tw] OR "thailandamide A"[tw] OR "Thalidomide"[tw] OR "Thiamphenicol"[tw] OR "thienamycin"[tw] OR "Thienamycins"[tw] OR "Thioacetazone"[tw] OR "thiobenzamide"[tw] OR "thiocarlide"[tw] OR "thiolactomycin"[tw] OR "thiomandelic acid"[tw] OR "Thiostrepton"[tw] OR "thymopoietin III"[tw] OR "tiamulin"[tw] OR "Ticarcillin"[tw] OR "ticarcillin-clavulanic acid"[tw] OR "Tigecycline"[tw] OR "tilmicosin"[tw] OR "Tinidazole"[tw] OR "Tobramycin"[tw] OR "Tobramycin, Dexamethasone Drug Combination"[tw] OR "tomaymycin"[tw] OR "Trimethoprim, Sulfamethoxazole Drug Combination"[tw] OR "triostin A"[tw] OR "Troleandomycin"[tw] OR "tsushimycin"[tw] OR "tulathromycin"[tw] OR "Tunicamycin"[tw] OR "Tylosin"[tw] OR "Tyrocidine"[tw] OR "Tyrothricin"[tw] OR "tyrothricin, benzalkonium chloride, benzocaine drug combination"[tw] OR "ubenimex"[tw] OR "ulifloxacin"[tw] OR "undecylprodigiosin"[tw] OR "vaborbactam"[tw] OR "Valinomycin"[tw] OR "Vancomycin"[tw] OR "VD 2085"[tw] OR "Vernamycin B"[tw] OR "Viomycin"[tw] OR "Virginiamycin"[tw] OR "ZINC03787097"[tw])

***AND*** (prophyla*[tw] OR "prevention and control"[Subheading] OR "Preventive Health Services"[Mesh] OR prevent*[tw])))

***AND*** ("Pancreaticoduodenectomy"[Mesh] OR "pancreaticoduodenectomy"[tw] OR pancreaticoduodenectom*[tw] OR "pancreatoduodenectomy"[tw] OR pancreatoduodenectom*[tw] OR "duodenopancreatectomy"[tw] OR duodenopancreatectom*[tw] OR "pancreatico duodenectomy"[tw] OR pancreatico duodenect*[tw] OR "duodeno pancreatectomy"[tw] OR duodeno pancreatectom*[tw] OR whipple procedure*[tw] OR whipple resect*[tw] OR whipple surger*[tw]))

Results demonstrated in Figure 1: 448 articles from five databases (November 10^th^ 2022)

**Appendix S2.** **CDC definition for superficial SSIs and OSIs**


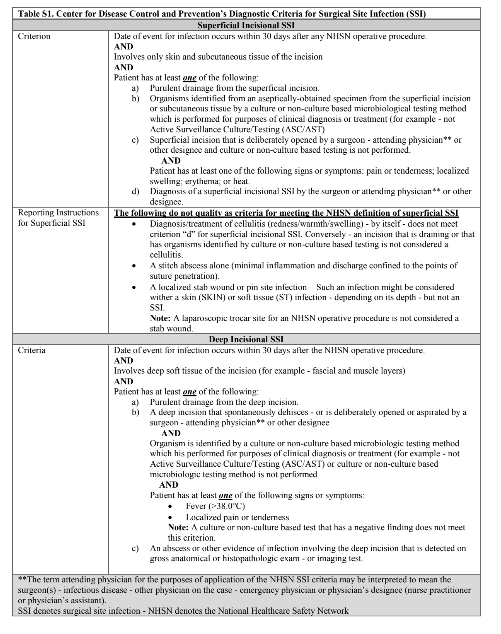


“Organ/space SSIs must meet the following criteria:

- Infection occurs within 30 days after the operative procedure if no implant is left in place or within 1 year if implant is in place *and*
- The infection appears to be related to the operative procedure and infection involves any part of the anatomy (e.g., organs or spaces) other than the incision opened or manipulated during the operative procedure, and at least one of the following is present:
  - 1. Purulent drainage from a drain that is placed through a stab wound* into the organ/space.
  - 2. Organisms isolated from an aseptically obtained culture of fluid or tissue in the organ/space.
  - 3. An abscess or other evidence of infection involving the organ/space on direct examination, during reoperation, or by histopathologic or radiologic examination.
  - 4. Diagnosis of an organ/space SSI by a surgeon or attending physician.”

**Supplementary Figures**

**Figure S1. OSI in patients with perioperative versus prolonged prophylaxis in all patients undergoing pancreatoduodenectomy (A) and in only patients who underwent preoperative biliary (B), calculated using the Mantel-Haenszel random-effects model.**


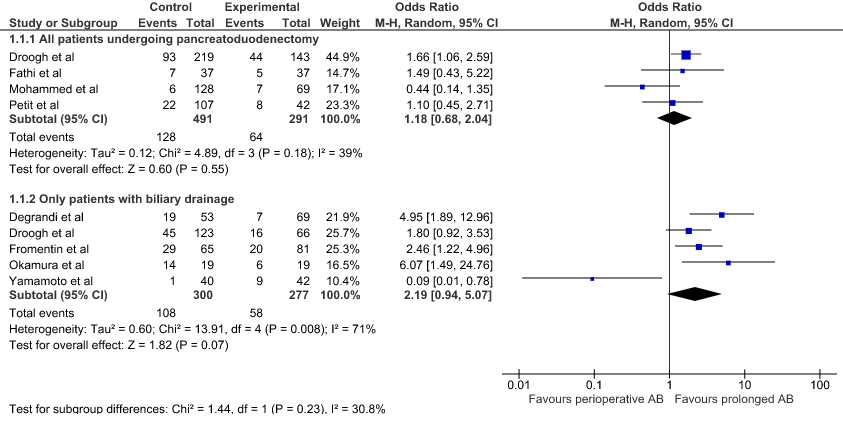


**A)**

**B)**

**Figures S2. Funnel plot OSI (A), superficial SSI (B), POPF (C), bacteraemia (D)**


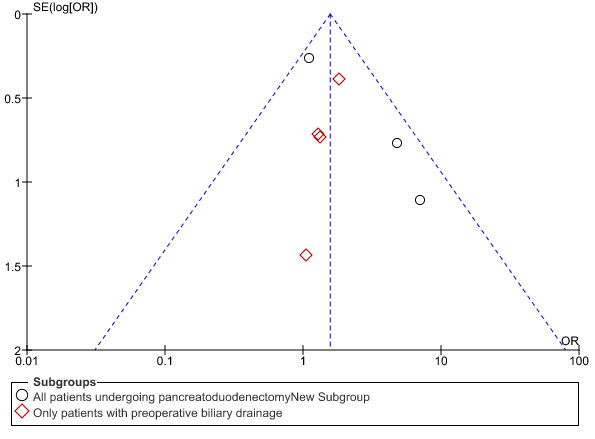

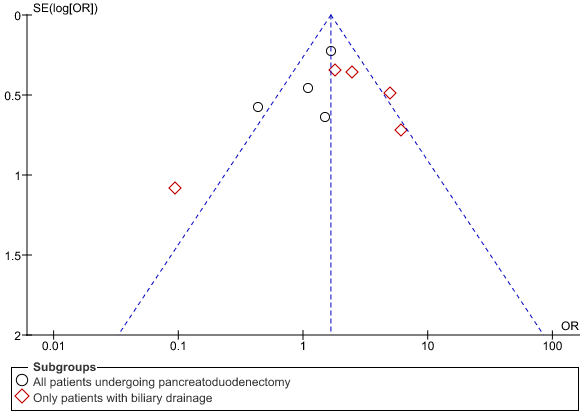
**A)**  **B)**

**C) D)**


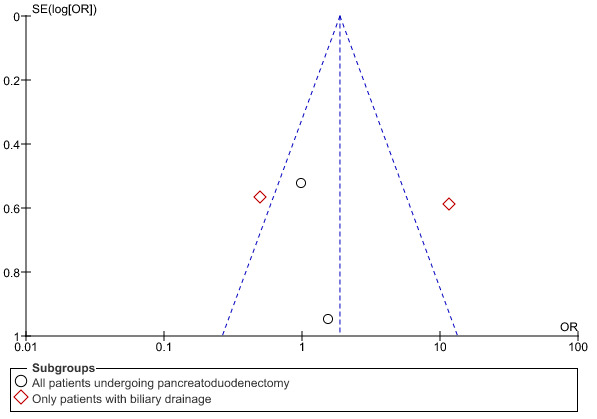

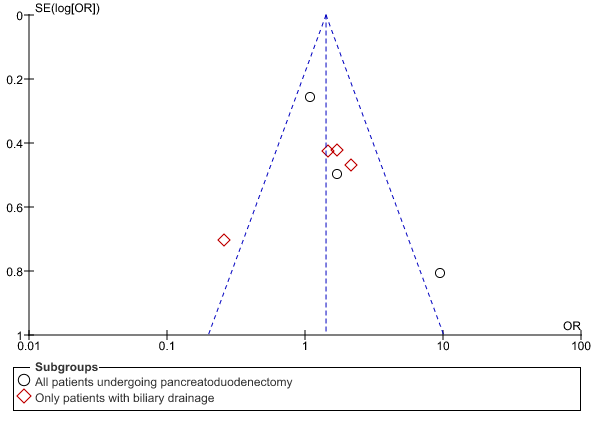


**Figures S3. Length of hospital stay in patients with perioperative versus prolonged prophylaxis in all patients undergoing pancreatoduodenectomy (A) and in only patients who underwent preoperative biliary (B), including funnel plot (C).**


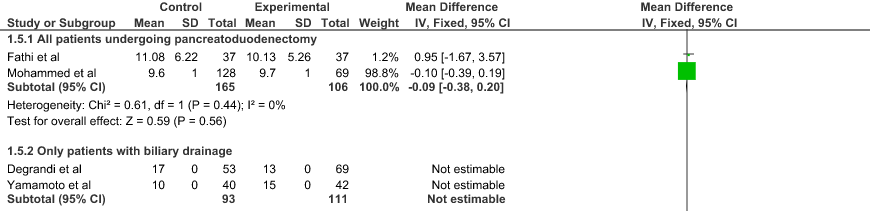


**A)**


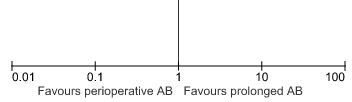


**C) Funnel plot**


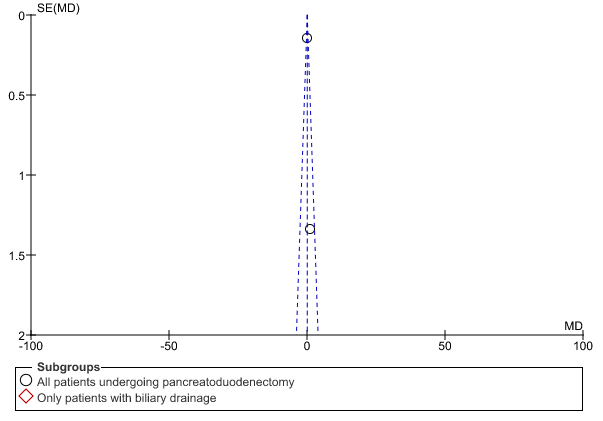

Supplement: znad213_Supplementary_Data [file znad213_supplementary_data.zip › Supplementary_Material.docx]
